# Survivin Regulates Bad Gene Expression by Binding to Its Promoter and Modulates Cell Cycle and Apoptosis in Esophageal Carcinoma Cell

**DOI:** 10.1155/2021/1384289

**Published:** 2021-01-05

**Authors:** Yan Chen, Shayahati Bieerkehazhi, Xiumei Li, Lili Ma, Waresijiang Yibulayin, Jihua Ran

**Affiliations:** ^1^Department of Biochemistry and Molecular Biology, School of Basic Medicine, Xinjiang Medical University, Xinjiang, China; ^2^Morphology Center, School of Basic Medicine, Xinjiang Medical University, Xinjiang, China; ^3^Department of Clinical Laboratory, The Fifth Affiliated Hospital of Xinjiang Medical University, Xinjiang, China; ^4^Department of Thoracic Surgery, The Affiliated Cancer Hospital of Xinjiang Medical University, Xinjiang, China; ^5^Clinical Laboratory Diagnostic Center, General Hospital of Xinjiang Military Region, Xinjiang, China

## Abstract

Esophageal cancer (EC) is the eighth most prevalent cancer and the sixth leading cause of cancer-related mortality worldwide. As an antiapoptotic and a proapoptotic protein, respectively, survivin and Bad play an important role in carcinogenesis of the most human cancers including EC. However, the regulatory relationships between them remain unclear. We sought to investigate the effects of survivin knockdown and overexpression on the expression of Bad gene, cell cycle progression, and apoptosis of esophageal carcinoma cell. The mRNA expression levels of survivin and Bad were determined in EC tissue samples. The knockdown and overexpression experiments were performed in ECA109 and KYSE450 cells via transfection with survivin overexpression and shRNA plasmids. A Bad overexpression experiment was conducted to confirm the biological effect on knockdown of survivin via modulating Bad expression. RT-qPCR and Western blot analysis were used to detect mRNA and protein expression, respectively. Cell cycle and apoptosis were analyzed by flow cytometry. The chromatin immunoprecipitation (ChIP) was conducted to determine the binding sites of survivin on the promoter of Bad gene. By analyzing the mRNA expression of survivin and Bad in 40 ESCC patient specimens, we found that the positive expression rate of survivin in tumor tissues (88%, 35/40) was remarkably high, compared with the distal nontumor tissues (48%, 19/40, *p* < 0.01). On the other hand, the positive expression rate of Bad in tumor tissues (70%, 28/40) was remarkably low, compared with the distal nontumor tissues (95%, 38/40, *p* < 0.01). Overexpression of survivin decreases Bad mRNA and protein expression and promotes transformation of cell cycle to S phase. Conversely, knockdown of survivin increases Bad mRNA and protein expression and induces cell cycle arrest and apoptosis. Bad overexpression inducing apoptosis of esophageal carcinoma cell shows the similar apoptotic effect with survivin knockdown. ChIP assays indicate that survivin directly binds to the Bad promoter region, diminishing the transcriptional activity of Bad. In conclusion, the result suggested that survivin regulates Bad gene expression by binding to its promoter and modulates cell cycle and apoptosis in esophageal carcinoma cell.

## 1. Introduction

Esophageal cancer (EC) is the eighth most prevalent cancer and the sixth leading cause of cancer-related mortality worldwide [1, 2]. It has two major histologic types including esophageal squamous cell carcinoma (ESCC) and adenocarcinoma. It is estimated that more than 480,000 new cases are diagnosed yearly [2] and more than 80% of esophageal cancers are ESCC [3]. Lacking of the sensitive method for early detection of ESCC, many patients with these tumors have adjacent invasion or distal metastases at the time of diagnosis. Despite recent advances in surgical techniques and chemoradiation, the prognosis of ESCC is relatively poor and the survival rate remains generally low [4]. Recently, an increasing highlight has been focused on survivin as an important marker for diagnosis and prognosis, a molecular target for therapeutic interventions, and a crucial mechanism for tumorigenesis [5–8]. Particularly, survivin overexpression has clinicopathological and prognostic significance in EC. A meta-analysis indicated that survivin expression seems to be associated with a worse prognosis of ESCC patients [9].

Survivin protein, a member of the inhibitor of apoptosis (IAP) family, is encoded by the BIRC5 gene in humans [10]. IAPs block apoptosis process by inhibiting the activation of caspases through direct binding to them. Survivin interacts with effector caspases (caspase-3 and 7), functioning as a negative regulator of apoptosis process [6]. Survivin has been extensively reported in various human cancers as a prognostic marker or a therapeutic target because of its important role in cell processes, such as apoptosis and cell division [5–8, 10]. However, there are rare reports on survivin as a transcriptional regulator of genes involving in tumorigenesis. Previously, we reported that survivin activates NF-*κ*B (nuclear factor kappa-B) p65 by regulating the expression levels of IKK*β* (inhibitor of nuclear factor *κ*B kinase subunit *β*) in esophageal cancer cell lines [11].

BH3-only protein Bad (Bcl-Xl/Bcl-2-associated death promoter homologue), a member of the Bcl-2 family, characters as a proapoptotic protein [12]. Dephosphorylated Bad translocates to mitochondrial membrane where it binds to and inactivates the antiapoptotic protein including Bcl-2 and Bcl-xl [13]. Phosphorylation of Bad at Ser112, Ser136, and Ser155 inhibits its proapoptotic activity in response to growth and survival signal [13]. Thereby, Bad plays a crucial role in connecting the cell survival signaling pathway and apoptosis signaling pathway. Clinical significance of Bad has been identified in many types of cancer. Both high expression and low expression of Bad are associated with outcomes of the patient with cancer [14–16].

In the present study, mRNA expression of survivin and Bad in 40 paired tumor tissues of ESCC patients was examined. Subsequently, the experiments of upregulation and downregulation of survivin were performed by infecting EC cell lines (KYSE450, ECA109) with overexpression and shRNA plasmids. A Bad overexpression experiment was conducted to confirm the biological effect on knockdown of survivin via modulating Bad expression. After transfection of 48 hours, mRNA and protein expression levels of survivin and Bad were examined. Meanwhile, apoptosis rate and cell cycle distribution were evaluated. Finally, in order to confirm the transcriptional regulation between survivin and Bad, ChIP assay was conducted to determine whether survivin binds to the promoter region of Bad gene. Our ﬁndings demonstrated that survivin regulates Bad gene expression by binding to its promoter and modulates cell cycle and apoptosis in EC cell lines.

## 2. Method and Materials

### 2.1. Tumor Tissue Specimens

Forty pairs of tumor and distal normal tissue samples were prospectively collected from surgically excised specimens of patients with ESCC at the Affiliated Cancer Hospital of Xinjiang Medical University (Urumqi, China) between July and December 2016. The tumor and distal tissues were frozen in liquid nitrogen immediately following resection. All patients in the current study did not receive chemotherapy or radiation therapy prior to surgery. The study was approved by the Ethics Committee of the Affiliated Cancer Hospital of Xinjiang Medical University. Written informed consent was provided by the families of all of the patients.

### 2.2. Cell Culture

ECA109 cell line was obtained from the Cell Bank of Xinjiang Medical University (Urumqi, China). KYSE450 cell line was purchased from the Cell Bank of the Chinese Academy of Sciences. ECA109 and KYSE450 cells were cultured in the RPMI-1640 medium (Gibco, Thermo Fisher Scientific, Inc., Waltham, MA, USA) supplemented with 10% heat-inactivated fetal bovine serum (FBS, Zhejiang Tianhang Biological Technology Co., Ltd., Zhejiang, China), 100 units/ml penicillin, and 100 *μ*g/ml streptomycin (Gibco, Thermo Fisher Scientific, Inc.) at 37˚C in a humidified 5% CO2 atmosphere.

### 2.3. GV142-Survivin Overexpression Plasmid Construction

The GV142 plasmid was purchased from GeneChem Co., Ltd. (Shanghai, China). For the GV142-survivin overexpression and GV142-control plasmid construction, GV227 (GeneChem Co., Ltd.) was used as the template, and the survivin polymerase chain reaction (PCR) primers used are presented in Table 1. The resulting PCR products were inserted into the GV142 vector between HindIII and XhoI sites, yielding GV142-survivin overexpression and GV142-control plasmids.

### 2.4. LV3-Survivin shRNA Plasmid Constructs

The LV3 vector was purchased from Shanghai GeneChem Co., Ltd. (Shanghai, China). The sequences of small shRNA targeting survivin were designed as follows: GAAAGTGCGCCGTGCCATCTTCAAGAGAGATGGCACGGCGCACTTTCTT. The sequences of negative control were designed as follows: GCGCGCACAATCTACGCTAGTTTCAAGAGAACTAGCGTAGATTGTGCGCGCTT. The sequences were inserted between the HindIII and XhoI sites of the LV3 vector chemically synthesized by Shanghai GeneChem Co., Ltd. The constructs were verified by DNA sequence analysis.

### 2.5. GV142-Bad Overexpression Plasmid Construction

In order to confirm the biological effect on knockdown of survivin via modulating Bad expression, we conducted a Bad overexpression experiment. GV142-Bad overexpression plasmid construction is similar to GV142-survivin overexpression plasmid construction as previously described. The Bad PCR primers used are also presented in Table 1.

### 2.6. Plasmid Transfection

Prior to electroporation, KYSE450 and ECA109 cells were ensured in exponential growth phase. The culture medium was removed and replaced with fresh serum-free Opti-MEM I medium. Cells were clicked and resuspended before centrifugation. After centrifugation, the supernatant was discarded. This step was repeated twice. 400 ul cell suspension was transferred into electroporation cuvette. Then, plasmid was added to cuvette. The cuvette was subjected to the electroporation (500 V, 15 ms, and square wave). After electroporation, the cells were transferred into a 6-well plate containing a complete medium to culture. Transfection efficiency was checked 24 hours after transfection by watching the glowing cell under fluorescence microscope because the plasmid contained the fluorescent protein gene.

### 2.7. Groups of Cells Infected with Plasmids

Cancer cells including KYSE450 and ECA109 were divided into three groups both in survivin overexpression and knockdown experiment. Groups in survivin overexpression are the UP group, NC group, and BC group. Cells in the UP group were transfected with GV142-survivin. Cells in the NC group were transfected with GV142-negative control. The BC group is blank control, and cells in the BC group were not treated with any plasmid during the electroporation. Groups in survivin knockdown are the KD group, NC group, and BC group. Cells in the KD group were transfected with LV3-survivin shRNA. Cells in the NC group were transfected with LV3-negative control. Cells in the BC group were not treated with any plasmid during the electroporation.

In the Bad overexpression experiment, cancer cells including KYSE450 and ECA109 were also divided into three groups. Groups in Bad overexpression are the UP_Bad group, NC group, and BC group. Cells in the UP_Bad group were transfected with GV142-Bad. Cells in the NC group were transfected with GV142-negative control. The BC group is blank control, and cells in the BC group were not treated with any plasmid during the electroporation.

### 2.8. RT-PCR for Analysis of mRNA from Patient's Tissue

Total RNA was isolated with TRIzol (Thermo Fisher Scientific, Inc.) following instructions provided by the manufacturer. RNA samples were quantified with ultraviolet spectrophotometer and severed as templates to generate cDNA.

### 2.9. Real-Time Quantitative PCR for Analysis of Cellular mRNA

48 hours after transfection, total cellular RNA extraction from cultured cell lines was performed using TRIzol (Thermo Fisher Scientific, Inc.) following instructions provided by the manufacturer. One *µ*g of total RNA extracted from the cells was subjected to reverse transcription. Maloney Murine Leukemia Virus Reverse Transcriptase (Promega Corporation, Madison, WI, USA) was used for cDNA synthesis. Specific primers were as follows: survivin, forward primer 5′-CCCTGCCTGGCAGCCCTTTC-3′ and reverse primer 5′-CTGGCTCCCAGCCTTCCA-3'; Bad, forward primer 5′-CAGAGTTTGAGCCGAGTGAGC-3′ and reverse primer 5′-CCCATCCCTTCGTCGTCCT-3'; caspase-3, forward primer 5′-GCTATTGTGAGGCGGTTGT-3′ and reverse primer 5′-AGCAGGGCTCGCTAACTC -3'; caspase-9, forward primer 5′-CGAACTAACAGGCAAGCA-3″ and reverse primer 5′-GCACCGACATCACCAAAT-3'; GAPDH, forward primer 5′-GGGAAACTGTGGCGTGAT-3′ and reverse primer 5′-AAAGGTGGAGGAGTGGGT-3'. Real-time PCR (RT-qPCR) was used to quantify the expression level of the survivin, Bad, caspase-3, and caspase-7 gene in the ESCC cell lines ECA109 and KYSE450 using the TaqMan® Fast Virus 1-Step Master Mix kit (Thermo Fisher Scientific, Inc.), according to the protocol supplied by the manufacturer. Amplification conditions were as follows: 2 min at 50˚C, 2 min at 95°C, 15 sec at 95°C, 15 sec at 55–60˚C, and 1 min at 72°C. The relative quantification transcript levels were calculated using the 2−ΔΔCq method. The experiments were performed in triplicate for each cell line, and results are presented as the mean ± standard deviation.

### 2.10. Western Blotting Assay

Forty-eight hours after transfection, attached and floating cells were harvested on ice. The cells were washed with cold PBS and subsequently lysed in cold RIPA lysis buffer (1 M Tris HCl (pH 7.4), 5M NaCl, 0.5 M ethylene glycol tetraacetic acid, 0.5 M EDTA, NP-40, 10% SDS (Wuhan Boster Biological Technology, Ltd., Wuhan, China), glycerine, 10 *µ*g/*µ*l aprotinin, 10 *µ*g/*µ*l leupeptin, 10 *µ*g/*µ*l pepstatin A, 10 mM phenylmethylsulfonyl fluoride, and double-distilled H_2_O) for 30 min on ice. Clear protein extracts were obtained by centrifugation at 18,407 xg for 15 min at 4°C. Protein concentrations were determined by Pierce BCA protein assay (Thermo Fisher Scientific, Inc.), and 20 mg of protein mixed with loading buffer was loaded per lane and separated by 10–15% polyacrylamide gels (Wuhan Boster Biological Technology, Ltd.). Proteins were transferred to PVDF membranes. Nonspecific binding was blocked by blocking with 5% nonfat dried milk (Sigma-Aldrich) in Tris-buffered saline and Tween-20 (TBST, Beijing Solarbio Science and Technology Co., Ltd., Beijing, China) at room temperature for 2 h. Membranes were incubated with the primary antibody overnight at 4°C. The primary antibodies include rabbit antisurvivin, anti-Bad, anti-Bad, anti-caspase-3, anti-caspase-7, and anti-GAPDH served as a loading control. Then, the membranes were washed three times with TBST at room temperature and incubated with appropriate horseradish peroxidase-linked goat anti-rabbit secondary antibodies at a dilution of 1 : 1,000 (cat. no. BA1054, Wuhan Boster Biological Technology, Ltd.) diluted in TBST for 1 h at room temperature. The immunoreactive bands were visualized using an Enhanced Chemiluminescence Detection Kit (Thermo Fisher Scientific, Inc.).

### 2.11. Cycle and Cell Apoptosis Analysis by Flow Cytometry

ECA109 and KYSE450 cells were directly incubated, at 37°C for 48 h, in 6-well plates and collected 48 h after transfection. Then, the cells were treated with the indicative reagent propidium iodide (PI) and Annexin V staining kit (BestBio Co. Ltd.). For the cell cycle analysis, the cells were washed with phosphate buffered saline (PBS) for 5 min and subsequently centrifugation at 900 g. The cells were collected and fixed in ice-cold 70% ethanol for 2 hours at 4°C, followed by treatment with 0.2 mg/ml RNase A (EMD Millipore) in PBS for 30 min at 37°C. PI was added (final concentration, 25 *μ*g/ml), and the cells were incubated for 30 min at 4°C in the dark prior to cell cycle analysis. Analysis of the samples was performed within 24 h. To determine the apoptosis rate, the transfected cells were washed twice with ice-cold PBS and resuspended in 195 *μ*l 1X Binding Buffer (EMD Millipore) to a concentration of 1x104 cells/ml. Annexin V (5 *μ*l) and PI were gently mixed with the cells and incubated for 15 min at room temperature in the dark. The dyes were washed out by centrifugation for 5 min at 94 xg, and the cells were resuspended in 190 *μ*l 1X Binding Buffer. PI staining solution (10 *μ*l) was gently mixed and incubated on ice and in the dark. The samples were analyzed within 1 h. All samples for the two assays consisted of 10,000 cells and were analyzed by fluorescence-activated cell sorting with a BD FACSMicroCount™ system (BD Biosciences, Franklin Lakes, NJ, USA). The experiments were performed independently in triplicate for each cell line.

### 2.12. Chromatin Immunoprecipitation Assay (ChIP)

To determine whether survivin binds to the Bad promoter region, ChIP assays were performed using the ChIP kit (EPIGENTEK, USA) according to the manufacturer's protocols. DNA and proteins in ECA109 and KYSE450 cells were cross-linked by treatment with 1% formaldehyde (Sigma-Aldrich) for 10 min. The cells were washed twice with 1X PBS, lysed, and sonicated to reduce DNA lengths to within the range of 200–1,000 bp. Immunoprecipitation was then performed using 4 *µ*g rabbit antibody against survivin to incubate. The group which incubated with normal mouse IgG served as the negative control. The group which incubated with 1 *µ*l anti-RNA polymerase II (dilution, 1 : 1,000) served as the positive control. The immune complexes were precipitated, eluted, reverse-crosslinked, and treated with proteinase K (Tiangen Biotech (Beijing) Co., Ltd., Beijing, China). The primers designed to amplify various regions of the human Bad gene promoters were as follows: region 1 (196 bp), 5′-GAGGTTCATAAGCGTCAAGGT-3′(forward) and 5′-GTATGGGCACAAGCGTCTC-3′(reverse); region 2 (252 bp), 5′-CCTTCGCCCGCAGTAATC-3' (forward) and 5′-CCTCGTCCGCATCCTTTT-3' (reverse); region 3 (431 bp), 5′-CTGGGCAAAGTAGAGGTTCAT-3′(forward) and 5′-TCCGTATTTATTTCCCTGGTC-3' (reverse); region 4 (490), 5′-CTGGGCAAAGTAGAGGTTCAT -3′(forward) and 5′-TCCGTATTTATTTCCCTGGTC-3' (reverse). The group which did not add any primers served as the primer blank control. PCR fragments were separated and visualized on 1.8% agarose gels stained with ethidium bromide (Shanghai Bioleaf Biotech Co., Ltd., Shanghai, China). All ChIP assays were performed independently in triplicate, and the most representative results are illustrated in the figures.

#### 2.12.1. Statistical analysis

All statistical analyses were performed with SPSS version 17.0 for Windows (SPSS, Inc., Chicago, IL, USA). Difference and correlation were analyzed by the *χ*2 test. *p* < 0.05 was considered statistically significant. Data (mRNA/protein levels, cell cycle, and cell apoptosis) were expressed as the mean ± standard deviation from three independent experiments. Data were analyzed by one-way analysis of variance followed by the LSD post hoc test used to compare mean differences in two groups. *p* < 0.05 was considered to indicate a statistically significant difference.

## 3. Result

### 3.1. Expression of Survivin and Bad in Esophageal Cancer Studied by RT-PCR

We studied 40 patients with ESCC. Tumor samples and paired distal normal tissues for mRNA expression of survivin and Bad were examined by RT-PCR. Survivin was expressed in the 35 of 40 tumor tissues, where expression rate was 88%, and it is expressed in the 19 of 40 normal tissues, where expression rate was 48%. The positive expression rate of survivin in tumor tissues was remarkably high, compared with the distal nontumor tissues (*p* < 0.01, Table 2). Bad was expressed in 28 of 40 tumor tissues, where expression rate was 70%, and it was expressed in the 38 of 40 normal tissues, where expression rate was 95% (Table 2). The positive expression rate of Bad in tumor tissues was remarkably low, compared with the distal nontumor tissues (*p* < 0.01, Table 2).

### 3.2. Overexpression of Survivin Decreases Bad mRNA and Protein Expression

**Figure 1:**
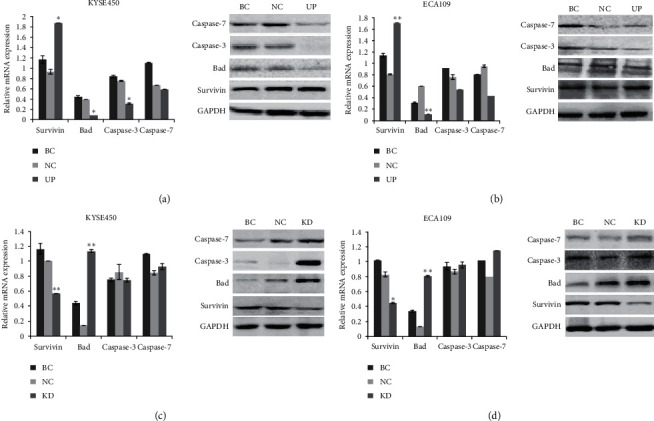
The effects of survivin overexpression and knockdown on the expression of Bad, caspase-3, and caspase-7 gene. The expressions of survivin, Bad, caspase-3, and 7 were determined by qRT-PCR and confirmed by Western blot. GADPH served as an internal and loading control. (a) When survivin was overexpressed in KYSE450 cells, Bad (^*∗*^*p* < 0.05) and caspase-3 (^*∗*^*p* < 0.05) were downregulated. (b) When survivin was overexpressed in ECA109 cells, Bad (^*∗∗*^*p* < 0.01) was downregulated. (c) When survivin was downregulated in KYSE450 cells, Bad (*p* < 0.01) was upregulated, but there were no significant differences in caspase-3 (*p* > 0.05) and caspase-7 (*p* > 0.05). (d) When survivin was downregulated in ECA109 cells, Bad (*p* < 0.01) was upregulated, but there were no significant differences in caspase-3 (*p* > 0.05) and caspase-7 (*p* > 0.05) (Fig 1), compared to BC and NC groups. Data are presented as the means ± standard deviation from triplicate experiments. BC: blank control; NC, UP, and KD: cells in these groups were transfected with negative control, overexpression, and shRNA plasmid, respectively. Statistically significant differences compared to BC and NC groups are indicated (^*∗*^*p* < 0.05, ^*∗∗*^*p* < 0.01).

To further explore the regulation relationship between survivin and proapoptotic factor Bad, we examined whether overexpression of survivin was able to modulate the expression of Bad and other apoptosis-associated proteins including caspase-3 and 7. Following the transfection of KYSE450 and ECA109 cells with GV142-survivin overexpression plasmid and controls, the mRNA levels of survivin, Bad, caspase-3, and 7 were examined by RT-qPCR. The results indicated that when survivin was overexpression in KYSE450 cells, Bad (*p* < 0.05, Figure 1(a)) and caspase-3 (*p* < 0.05) were downregulated but caspase-7 (*p* > 0.05, Figure 1(a)). When survivin was overexpression in ECA109 cells, only Bad (*p* < 0.05, Figure 1(b) was downregulated, compared to blank and negative control (Figure 1). Immunoblotting confirmed that overexpression of survivin can downregulate expression of Bad protein both in KYSE450 and ECA109 cells (Figure 1).

### 3.3. Knockdown of Survivin Increases Bad mRNA and Protein Expression

Following the transfection of KYSE450 and ECA109 cells with LV3-survivin shRNA plasmid and controls, the mRNA levels of survivin, Bad, caspase-3, and caspase-7 were examined by RT-qPCR. The results indicated that when survivin was downregulated in KYSE450 cells, Bad (*p* < 0.01) was upregulated, but there were no significant differences in caspase-3 (*p* > 0.05) and caspase-7 (*p* > 0.05) (Figure 1). When survivin was downregulated in ECA109 cells, Bad (*p* < 0.01) was upregulated, but there were no significant differences in caspase-3 (*p* > 0.05) and caspase-7 (*p* > 0.05) (Figure 1), compared to BC and NC groups (Figure 1). Immunoblotting confirmed that downexpression of survivin can upregulate expression of Bad protein both in KYSE450 and ECA109 cells (Figure 1).

### 3.4. Survivin Overexpression Promotes Transformation of Cell Cycle to S Phase

**Figure 2:**
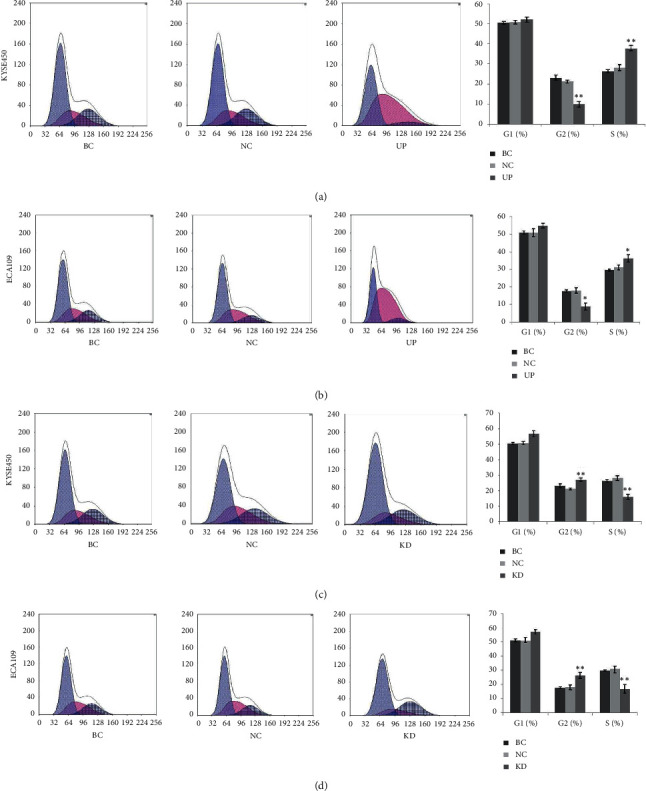
The effects of survivin overexpression and knockdown on the cell cycle progression of esophageal carcinoma cell. (a) The UP groups exhibited a decreased number of cells in the G2 phase but an increased number of cells in the S phases in KYSE450 cells. (b) The UP groups exhibited a decreased number of cells in the G2 phase but an increased number of cells in the S phases in ECA109 cells. (c) The KD groups exhibited an increased number of cells in the G2 phase but a decreased number of cells in the S phases in KYSE450 cells. (d) The KD groups exhibited an increased number of cells in the G2 phase, but a decreased number of cells in the S phases in ECA109 cells. Data are presented as the means ± standard deviation from triplicate experiments. BC: blank control; NC, UP, and KD: cells in these groups were transfected with negative control, overexpression, and shRNA plasmid, respectively. Statistically significant differences compared to BC and NC groups are indicated (^*∗*^*p* < 0.05, ^*∗∗*^*p* < 0.01).

**Figure 3:**
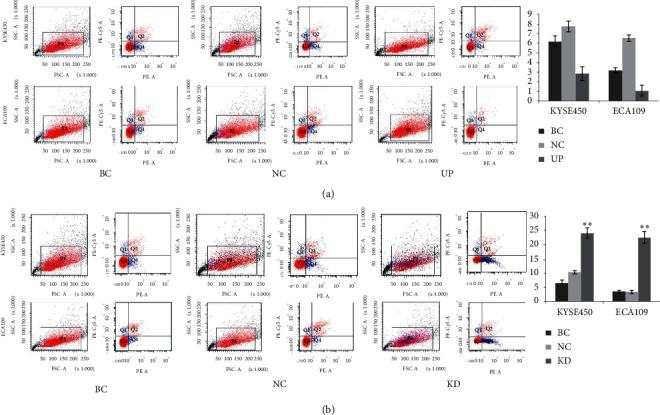
The effects of survivin overexpression and knockdown on the apoptosis of esophageal carcinoma cell. (a) There was not significantly alteration of apoptotic rate among UP, BC, and NC groups both in KYSE450 and ECA109 cells. (b) Apoptotic rate of the KD group was significantly higher compared with the BC and NC groups. Data are presented as the means ± standard deviation from triplicate experiments. BC: blank control; NC, UP, and KD: cells in these groups were transfected with negative control, overexpression, and shRNA plasmid, respectively. Statistically significant differences compared to BC and NC groups are indicated (^*∗*^*p* < 0.05, ^*∗∗*^*p* < 0.01).

To explore the effects of survivin overexpression on the viability of esophageal cancer cell, flow cytometry was adopted to detect alterations in cell cycle progression and apoptosis following survivin overexpression. In KYSE450 cell lines, cytometry showed that the proportions of cells in the G1 phase among the BC, NC, and UP groups were 50.52 ± 0.67%, 50.71 ± 1.01%, and 52.09 ± 1.27%, respectively. For these groups, the proportions of cells in the G2/M phase were 23.11 ± 1.23%, 21.20 ± 0.63%, and 9.96 ± 1.38%, respectively, and the proportions of cells in the S phase were 26.37 ± 0.80%, 28.09 ± 1.47%, and 37.85 ± 1.26%, respectively. Compared with the BC and NC groups, the proportion of cells in S phase in the UP group was significantly increased (*p* < 0.01, Figure 2(a)), whereas the ratio of cells in the G2/M phase was significantly decreased (*p* < 0.01, Figure 2(a)). However, there were no significant differences between the BC and NC groups. The significant alteration of apoptotic rate was not found among the UP (24.01 ± 1.75%), BC (6.42 ± 0.95%), and NC (10.23 ± 0.56%) groups (*p* > 0.05, Figure 3(a)). In ECA109 cell lines, approximately similar results of cell cycle progression and apoptosis were observed (Figures 2(b) and 3(a)). These results suggested that survivin overexpression promotes transformation of cell cycle to S phase.

### 3.5. Survivin Knockdown Induces Cell Cycle Arrest and Apoptosis

In KYSE450 cell lines, cytometry showed that the proportions of cells in the G1 phase among the BC, NC, and KD group were 50.52 ± 0.67%, 50.71 ± 1.01%, and 56.83 ± 1.96%, respectively. For these groups, the proportions of cells in the G2/M phase were 23.11 ± 1.23%, 21.20 ± 0.63%, and 27.13 ± 1.09%, respectively, and the proportions of cells in the S phase were 26.37 ± 0.80%, 28.09 ± 1.47%, and 16.04 ± 1.63%, respectively. Compared with the BC and NC groups, the proportion of cells in S phase in the KD group was significantly decreased (Figure 2(c), *p* < 0.01), whereas the ratio of cells in the G2/M phase was significantly increased (Figure 2(c), *p* < 0.01). Apoptotic rate of the KD group (24.01 ± 1.75%) was significantly higher compared with the BC (6.42 ± 0.95%) and NC (10.23 ± 0.56%) groups (*p* < 0.01) (Figure 3(b)). However, there were no significant differences between the BC and NC groups. In ECA109 cell lines, approximately similar results of cell cycle progression and apoptosis were observed (Figures 2(d) and 3(b)). These results suggested that survivin knockdown induces cell cycle arrest and apoptosis in esophageal carcinoma cell.

### 3.6. Bad Overexpression Induces Apoptosis of Esophageal Carcinoma Cell

**Figure 4:**
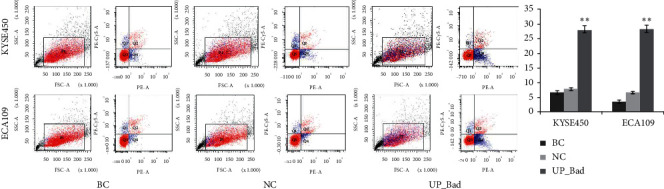
The effect of Bad overexpression on the apoptosis of esophageal carcinoma cell. Apoptotic rate of the UP_Bad group was significantly higher compared with the BC and NC groups. Data are presented as the means ± standard deviation from triplicate experiments. BC: blank control; NC, UP, and UP_Bad: cells in these groups were transfected with negative control and overexpression plasmid, respectively. Statistically significant differences compared to BC and NC groups are indicated (^*∗*^*p* < 0.05, ^*∗∗*^*p* < 0.01).

In KYSE450 cell lines, apoptotic rate of the UP_Bad group (27.90 ± 1.51%) was significantly higher compared with the BC (6.73 ± 0.59%) and NC (7.87 ± 0.35%) groups (*p* < 0.01) (Figure 4). However, there were no significant differences between the BC and NC groups. In ECA109 cell lines, approximately similar results of apoptosis were observed (Figure 4). Bad overexpression inducing apoptosis of esophageal carcinoma cell shows the similar apoptotic effect with survivin knockdown.

### 3.7. Survivin Binds to Bad Promoter and Regulates Bad mRNA Expression in KYSE450 and ECA109 Cell Lines

**Figure 5:**
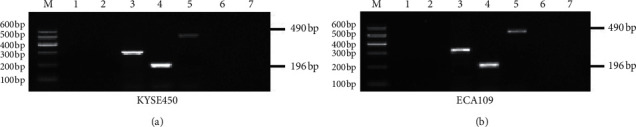
The representative PCR results of recovery DNA fragments from ChIP experiment in KYSE450 and ECA109 cell lines. The positive results of amplified fragment in the 4^th^ and 5^th^ lanes of agarose gel indicated that there were bind sites of survivin protein in the promoter region of Bad gene. 1^st^ lane of agarose gel: primer blank control, the group which did not add any primers. 2^nd^: negative control, the group which incubated with normal mouse IgG. 3^rd^: positive control, the group which incubated with 1 *µ*l anti-RNA polymerase II (dilution, 1 : 1,000). 4–7^th^: 4 primers designed to amplify various regions of the human Bad gene promoter.

A ChIP assay was performed to further confirm the direct interaction between survivin and the Bad promoter regions. Chromatin samples were incubated with anti-survivin antibody, anti-RNA polymerase II (positive control), and normal mouse IgG (negative control). The recovery DNA fragments from ChIP experiment in KYSE450 and ECA109 cell lines were subjected to PCR with promoter-specific primers for Bad. Four primers were designed to amplify various regions of the human Bad gene promoter. The group which did not add any primers served as primer blank control. The positive results of amplified fragment in the 4^th^ and 5^th^ lanes of agarose gel indicated that there were bind sites of survivin protein in the promoter region of Bad gene (Figure 5). These findings indicate that survivin directly binds to the Bad promoter region, diminishing the transcriptional activity of Bad.

## 4. Discussion

As an antiapoptotic and a proapoptotic protein, respectively, survivin and Bad play an important role in carcinogenesis of most human cancers. A number of studies have indicated that survivin is highly expressed in different cancer cells and primary tumor biopsy samples, but it is present at low levels or is completely absent in healthy cells and tissues [6]. However, expression of Bad is downregulated in many tumor tissues [16]. Our data showed that the mRNA level of survivin was increased, while the mRNA level of Bad was decreased in ESCC tissues compared to normal tissues.

Based on their expression characteristics in ESCC samples and the relevant literature reports, we speculate that there may be regulatory relationship between survivin and Bad. In order to test this hypothesis, we conducted a series of experiments to manipulate expression of survivin by transfecting KYSE450 and ECA109 cells with survivin expression vector and survivin. We measured the mRNA and protein expression of Bad, caspase-3, and caspase-7 in KYSE450 and ECA109 cells after transfection of the survivin expression vector and survivin. Western blot and qPCR analyses demonstrated that downregulation of survivin induced increases in Bad mRNA and protein levels in both cell lines, whereas overexpression of survivin resulted in decreased Bad mRNA and protein. However, there were not the same effects in caspase-3 and 7. It indicted that survivin might not regulate the expression of caspase-3 and 7 but binds directly to them, inhibiting the caspase-mediated cascade leading to apoptosis. Meanwhile, the biology behaviors of two cell lines including apoptosis rate and cell cycle distribution were also evaluated. Cytometry showed that survivin overexpression promotes transformation of cell cycle to S phase and knockdown induces cell cycle arrest and apoptosis. This result was consistent with our previous study and literature reports [11].

These findings made us to suspect the putative roles for survivin as a transcription regulator for Bad. A ChIP assay was conducted to further confirm the direct interaction between survivin and the Bad promoter. The ChIP assay indicated that survivin directly binds to the Bad promoter region, diminishing the transcriptional activity of Bad. According to our best knowledge, this is the first study to find that survivin as a transcriptional regulator regulates the expression of Bad. The result suggested that survivin is a novel transcriptional regulator of Bad. Survivin regulates Bad gene expression by binding to its promoter and modulates cell cycle and apoptosis in esophageal carcinoma cell.

## 5. Conclusions

In conclusion, our study demonstrated that the mRNA level of survivin was increased, while the mRNA level of Bad was decreased in ESCC tissues compared to normal tissues. Downregulation of survivin induced increases in Bad mRNA and protein levels in KYSE450 and ECA109 cell lines, whereas overexpression of survivin resulted in decreased Bad mRNA and protein. Bad overexpression inducing apoptosis of esophageal carcinoma cell shows the similar apoptotic effect with survivin knockdown. Survivin overexpression promotes transformation of cell cycle to S phase, and knockdown induces cell cycle arrest and apoptosis. A ChIP assay confirmed that the direct interaction between survivin and the Bad promoter. The result suggested that survivin is a novel transcriptional regulator of Bad. Survivin regulates Bad gene expression by binding to its promoter and modulates cell cycle and apoptosis in esophageal carcinoma cell.

## Figures and Tables

**Table 1:** Primers for GV142-survivin and GV142-Bad cDNA synthesis.

Gene	Primers 5′⟶3′		Product (bp)
	Forward	Reverse	
Survivin	TGCCAAGCTTATGGGTGCCCCGACGTTGC	TCCGCTCGAGTATCCATGGCAGCCAGCTGCTC	447
Bad	TCCAACTTTGTGCCAAGCTTATGTTCCAGATCCCAGAG	AATGCCAACTCTGTCTCGAGTCTGGGAGGGGGCGGAGCTTC	545

**Table 2:** The expression of survivin and Bad mRNA in tumor and distal normal tissue (*n* = 40).

	Tumor tissue		Normal tissue		*χ* ^2^	*p*
	(+)	(−)	(+)	(−)		
Survivin	35	5	19	21	14.587	≤0.001
Bad	28	12	38	2	8.658	0.003

## Data Availability

The data used to support the findings of this study are included within the article.
